# Effects of a physical activity and nutrition program in retirement villages: a cluster randomised controlled trial

**DOI:** 10.1186/s12966-017-0543-6

**Published:** 2017-07-11

**Authors:** Jonine Jancey, Anne-Marie Holt, Andy Lee, Deborah Kerr, Suzanne Robinson, Li Tang, A.S. Anderson, Andrew Hills, Peter Howat

**Affiliations:** 1 Collaboration for Evidence, Research and Impact in Public Health, School of Public Heath, Curtin University, GPO Box U1987, Perth, WA 6845 Australia; 2 School of Public Health, Curtin University, GPO Box U1987, Perth, WA 6845 Australia; 3 Chinese Evidence-based Medicine Center, West China Hospital, Sichuan University, No. 37 Guoxue Alley, Chengdu, Sichuan Province 610041 China; 4 Centre for Public Health Nutrition Research, Division of Cancer Research, Ninewells Medical School, Dundee University, Level 7, Mailbox 7, Dundee, DD1 9SY UK; 5 University of Tasmania, 41 Charles St, Launceston TAS, Launceston, TAS 7250 Australia

**Keywords:** Retirement villages, Physical activity, Strength exercise, Walking, Fruit and vegetable intake, Weight management

## Abstract

**Background:**

This cluster randomised controlled trial aimed to determine if a 6- month home-based intervention could improve the physical activity and dietary behaviours of adults aged 60 to 80 years living in retirement villages located in Perth, Western Australia.

**Methods:**

Participants (*n* = 363) from 38 retirement villages were recruited into the trial and allocated to the intervention (*n* = 197: 17 sites) or control (*n* = 166: 21 sites) group and were blinded. Previously validated instruments-Fat and Fibre Barometer and International Physical Activity Questionnaire, along with anthropometric measures (weight, height, waist and hip circumferences) and blood pressure were collected at baseline and 6 ﻿-month time period. Comparisons between intervention and control groups were undertaken pre- and post- intervention using univariate chi-square and t-tests. Multi-level mixed regression analyses were then conducted to ascertain the effects of the intervention on changes in the outcome variables over time and between groups.

**Results:**

A total of 139 (70.5%) intervention and 141 (84.9%) control group participants completed the program and post-test assessments. The intervention group demonstrated significant increases in time (80 min more per week on average) devoted to moderate-intensity physical activity, engagement in strength exercises (from 23.7% to 48.2%), frequency of fruit consumed as well as fat avoidance and fibre intake scores, in addition to a 0.5 kg mean reduction in weight post program, whereas no apparent changes were observed in the control group. Mixed regression results further confirmed statistically significant improvements in weight loss (*p* < 0.05), engagement in strength exercises (*p* < 0.001) and fruit intake (*p* = 0.012) by the intervention participants at post-test relative to their controls.

**Conclusions:**

Retirement offers a time to reassess lifestyle, and adopt positive health enhancing physical activity and dietary behaviours. This intervention was successful in improving weight, engagement in strength exercises, increasing levels of moderate-intensity physical activity and consumption of fruit among retirement village residents. Further investigation is needed on how to better engage retirement village managers in such programs.

**Trial registration:**

Australia and New Zealand Clinical Trial Registry (ACTRN12612001168842) registered November 2, 2012.

**Electronic supplementary material:**

The online version of this article (doi:10.1186/s12966-017-0543-6) contains supplementary material, which is available to authorized users.

## Background

As with other developed countries, Australia’s population is aging, with the proportion of adults aged over 65 predicted to increase to 25% by the year 2025 [1]. Worldwide, older adults are currently amongst the least physically active population group [2], with almost 50% of Australians aged over 60 years not meeting the recommended physical activity guidelines [1]. In addition, Australians consume a diet high in saturated fat, sugar and salt and low in fibre, fruit and vegetables [3], with between 8 and 10% of those aged 65–74 years not meeting the recommended intake for fruit and vegetables [1, 4]. This sedentary lifestyle combined with a less than optimal diet contributes to the increasing proportion of older adults being overweight and obese (more than 60%) [5].

Participation in regular physical activity, both aerobic and resistance training, along with the consumption of a healthy diet can counter functional decline and the associated chronic disease [6, 7]. Combining physical activity with dietary management can build muscle mass, increase metabolic rate while contributing to weight loss [4, 8]. Evidence also supports longer-term adherence to programs that are multi-component, such as a combination of physical activity and nutrition, as this type of program is more challenging and less repetitious [9]. However, reaching and motivating people to increase their level of physical activity and improve their dietary intake can be demanding, and even more so when these habits are well established, as with older adults.

In Australia, ‘retirement village’ refers to a range of housing types where adults live independently. Retirement villages are becoming an increasingly popular residential choice for older age groups [10]. An estimated 5.7% of Australian adults aged 65 years and over reside in over 2000 retirement villages, and demand for this type of accommodation is projected to rise to 7.5% in the next decade [10–13].

Retirement villages target functionally mobile and independent older adults, requiring no or very low level of domiciliary care [13–16]. The communities are usually ‘gated’ with housing purpose-built for an older population group, which offer a range of accommodation options, such as group housing and independent living units (ILUs) that are usually apartments or villas. Although there are often recreational and social facilities available [17, 18], the older adults residing in retirement villages tend to have sedentary lifestyles [12, 19, 20]. This makes retirement villages an ideal setting to target those with low levels of physical activity and less than optimum dietary behaviours [13, 21], as despite the inclusion of light recreation or therapy-based activities such as falls prevention [12], these activities offered to residents are poorly attended. [13, 21].

Retirement is a major life change that provides an opportunity for older adults to modify their eating and activity behaviours [22]. However, to date few randomised controlled trials have been undertaken [15, 23] to understand the impact of tailored physical activity and nutrition programs for older adults in retirement villages. This is surprising, considering the steady increase in the aging population and demand for this type of accommodation [10, 24, 25]. This study presents the results of a physical activity and nutrition intervention for adults aged 60 to 80 years living in retirement villages located in Perth, Western Australia. This study aimed to determine changes in retirement village residents’ levels of physical activity, dietary behaviours and anthropometry of the intervention group participants at the end of the 6- month intervention period, in comparison to the control group participants.

## Methods

### Study design

**Fig. 1:**
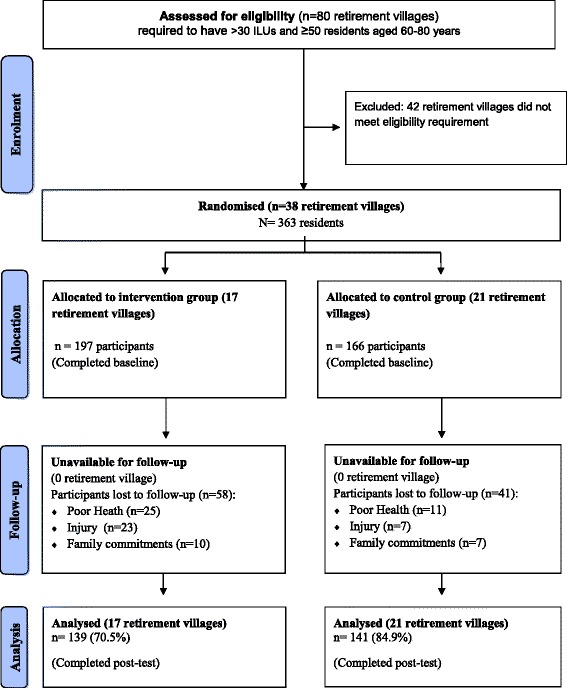
CONSORT flow chart of recruitment process

The protocol for this trial has been described in detail previously [10], in accordance with the Consolidated Standards of Reporting Trial (CONSORT) Statement (see Fig. 1 for Consort flow chart and Additional file 1 for the Consort Check list). This study was a cluster randomised controlled trial of a 6-month individual physical activity and nutrition intervention. Data were collected from the intervention and control participants at baseline and post-test (6-month time period). Ethical approval of the study was obtained from the Curtin University Human Research Ethics Committee (approval number HR 128/2012). Trial registration was lodged with the Australia and New Zealand Clinical Trial Registry (ACTRN12612001168842). All participants were informed of the study objectives, their rights and provided written informed consent prior to being included in the baseline data collection.

### Intervention

The retirement village physical activity and nutrition intervention program was guided by Social Cognitive Theory and Motivational Interviewing. The six-month intervention program was designed to support participants through personal goal setting, monitoring and feedback on progress, skill building as well as the provision of social support via group participation [26]. The program incorporated a number of educational resources (booklet, calendar, exercise chart, resistance bands and bi-monthly newsletters) based on the National Physical Activity and Dietary Guidelines [4, 27], and trained program ambassadors of a similar age. The program ambassadors were responsible for two face-to-face meetings whereby they introduced the program, and distributed and explained the program resources. This face-to- face introduction discussed goal setting, included a demonstration of the exercise program and responded to any questions. This was then followed-up by regular telephone contact by the program ambassadors. The frequency of the telephone contact was tailored to the needs of participants, which varied between weekly to monthly contact depending on individual preference, as was the motivational interviewing upon agreement with the participants. Motivational interviewing by these ambassadors via telephone contact supported participant goal setting, adherence and program sustainability [28]. Full details of the intervention have been reported elsewhere [12].

### Procedure

The study was conducted within a 75-km radius of metropolitan Perth, the capital of Western Australia. Retirement villages with over 30 ILUs were eligible for inclusion if they had at least 50 residents aged between 60 and 80 years. Initial contact was made with the village management and resident committees via telephone and followed up with an email describing the project and its aims. Upon agreement, the retirement village residents were informed of the project through an onsite information session promoted by the managers, and/or a reply paid postcard placed in their letterbox. The postcard explained the program, provided the researchers’ contact details and invited residents to return the postcard if they were interested in being involved in the program. This recruitment procedure had been used successfully in other settings with the same age group [29, 30]. Once informed consent was obtained, participants completed the Physical Activity Readiness Questionnaire (PARQ) at baseline [31].

### Participants

The 38 retirement villages located over a 75 km radius to avoid contamination, were randomly allocated to control (21 sites) or intervention (17 sites) groups using a table of random numbers, while taking into account the relative size of these retirement villages and the expected lower response rate of the controls. There were 1680 residents in total. From the 583 blinded residents who registered to participate in the study, 363 (intervention *n* = 197, control *n* = 166) met the selection criteria (reported undertaking less than 150 min of moderate-intensity physical activity per week, on no special diet and not participating in any other physical activity program).

### Measurement instruments

The International Physical Activity Questionnaire-Short Version [32] enabled collection of information on physical activity (walking, moderate- and vigorous-intensity activity) and sitting time in minutes per week. Definitions and examples of moderate- and vigorous-intensity physical activity were provided on the paper based self-complete questionnaire. Muscle strength exercise questions were taken from the American Heart Association guidelines [33].

Dietary habits were assessed via the validated Fat and Fibre Barometer [34]. This questionnaire contains 20 food behaviour items to assess an individual’s fat-related food intake (fried foods, dairy foods, meat, chicken and butter) and fibre-related food intake (whole grain foods, fruit and vegetables). Response values for each item range from 1 to 5, with ‘1’ representing food behaviour associated with the high fat intake or low fibre intake, to ‘5’ representing the low fat or high fibre intake. Fat and fibre scores were calculated by summing the scores from the corresponding fat and fibre foods consumed. Individual items on frequency of fruit and vegetables servings (servings were defined and depicted in the questionnaire), were also recorded.

Guided by a measurement protocol, anthropometric measurements were undertaken by a trained researcher and included height, weight, waist and hip circumferences using a portable stadiometer, calibrated electronic scale, and tape measure, respectively. Blood pressure was measured using an Omron M5–1 electronic sphygmomanometer. A mean value was obtained after for all three consecutive measurements. All measurements were taken at baseline and post-intervention (6- months) following standard protocols and guidelines. Demographic information recorded included gender, age, education level, relationship status, smoking status and presence of co-morbidities.

### Statistical analysis

Descriptive statistics were used to summarise the baseline demographic, health and lifestyle characteristics. Comparisons between intervention and control groups were undertaken across the two time points using independent samples and paired t-tests for continuous variables and chi-square test for categorical variables.

Co-primary outcomes of interest were physical activity level (for walking, sitting time, moderate- and vigorous-intensity activity and strength exercises) and dietary intake behaviours (fruit intake, vegetables intake, fibre intake, fat intake and fat avoidance), along with a host of secondary outcomes that included anthropometric status (weight, body mass index (BMI), waist-to-hip ratio (WHR)) and blood pressure. In the presence of many zeros, strength exercise and vigorous physical activity were dichotomised to indicate participation status (yes; no), whereas walking, sitting time and moderate-intensity physical activity remained as continuous variables (minutes per week). In terms of dietary behaviours, consumption of at least two servings of fruit (vegetables) on three to seven days per week was classified as frequent intake, and otherwise as infrequent. The fibre intake, fat intake and fat avoidance scores were calculated by summing individual item scores (range 1–5) for all corresponding applicable questions; the sum of each component was then divided by the number of applicable items.

To accommodate the correlation of repeated pre- and post- measures from the same person and the clustering of observations within retirement villages, multilevel mixed regression models with random effects (participants and retirement villages) [35] were fitted to assess the intervention effect on changes in outcome variables over time, while accounting for the influence of potential confounders (age, gender, height, education level, relationship status, smoking status, and presence of co-morbidity). All binary outcomes (strength exercise, vigorous-intensity physical activity, frequent fruit intake and frequent vegetable intake) were analysed using logistic mixed regression models, whereas linear mixed regressions were applied to anthropometric outcomes (weight, BMI, WHR), blood pressure, walking time, fibre intake, fat intake and fat avoidance scores. The walking variable was logarithmicly transformed prior to regression analysis due to its positively skewed distribution. Gamma mixed regression was considered appropriate for modelling the highly skewed sitting variable and moderate activity [26]. All statistical analyses were performed in the SPSS Statistical Package Version 22.0 [36]. Sample size calculations are reported elsewhere [10].

## Results

Thirty-eight (47.5%) of the 80 eligible retirement villages agreed to participate in the study. The retirement village on-site information sessions were the preferred and most effective mechanism to promote the program and recruit residents (66.4%), with the postcard distribution recruitment method being less effective (33.6%). Of the 363 residents who entered the program (intervention: *n* = 197, control: *n* = 166), 280 (intervention: *n* = 139, control: *n* = 141) remained at post-test, resulting in an overall retention rate of 77.7% (intervention: 70.5%, control: 80.9%). Of the 83 participants who withdrew, 43.4% (intervention: *n* = 25, control: *n* = 11) nominated poor health, 36.1% (intervention: *n* = 23, control: *n* = 7) were due to injury and 20.5% (intervention: *n* = 10, control: *n* = 7) because of family commitments (See Fig. 1).

The average age of the 280 program completers was 72 (SD 5.2) years, with a mean height of 1.6 (SD 0.1) metres. Nearly half had attained tertiary education (48.9%). The majority of participants were female (74.6%), had never smoked (61.1%) and experienced health conditions common to this age group (85.4%). Compared to controls, the intervention participants tended to live with a partner (*p* = 0.024). There were no statistically significant differences in demographic characteristics between those who completed the study (*n* = 280) and those who dropped out (*n* = 83). Table 1 presents the characteristics of the sample at baseline.

**Table 1:** Baseline characteristics of intervention participants and controls

Variables	Tota (*n* = 280)	Intervention group (*n* = 139)	Control group (*n* = 141)	*p* ^a^
Gender				0.449
Female	209 (74.6%)	101 (72.7%)	108 (76.6%)	
Male	71 (25.4%)	38 (27.3%)	33 (23.4%)	
Education level				0.326
Secondary school or below	143 (51.1%)	77 (55.4%)	66 (46.8%)	
Trade certificate/diploma	57 (20.4%)	27 (19.4%)	30 (21.3%)	
University	80 (28.5%)	35 (25.2%)	45 (31.9%)	
Relationship status				0.024
No partner	103 (36.8%)	42 (30.2%)	61 (43.3%)	
With partner	177 (63.2%)	97 (69.8%)	80 (56.7%)	
Smoking status				0.827
Never	171 (61.1%)	84 (60.4%)	87 (61.7%)	
Former/current	109 (38.9%)	55 (39.6%)	54 (38.3%)	
Co-morbidity ^b^				0.116
No	41 (14.6%)	25 (18.0%)	16 (11.3%)	
Yes	239 (85.4%)	114 (82.0%)	125 (88.7%)	
Age: mean (SD) years	72 (5.2)	72.71 (5.02)	71.88 (5.39)	0.186
Height: mean (SD) m	1.6 (0.1)	1.63 (0.09)	1.64 (0.10)	0.504

*SD* standard deviation

^a^Chi-square or *t*-test between intervention and control groups

^b^Presence of at least one of eight common health conditions

### Changes in physical activity

No significant differences were recorded in mean walking, sitting time (*p* > 0.05) and prevalence of vigorous-intensity activity (*p* > 0.10) in both groups, but a significant improvement in moderate-intensity activity was evident in the intervention group, an increase of 80-min per week on average, in contrast to only 8-min per week in the controls. Similarly, intervention participants exhibited significantly higher levels of engagement in strength exercise from baseline (23.7%) to post-program (48.2%) (*p* < 0.001) compared to a small increase (2%) in the control group (*p* = 0.693). Furthermore, significant differences were found in the time devoted to moderate-intensity physical activity (*p* = 0.004) and the prevalence of strength exercise (*p* = 0.002) between the two groups at 6- months.

### Changes in dietary behaviours

Although both groups reported similar fruit intake at baseline, the intervention participants showed a significant increased intake than controls post-program (*p* = 0.007). The intervention group also showed a significant increase in mean fibre (*p* = 0.006) and significant decrease in fat intake scores (*p* < 0.001). As expected, there was little change in dietary habits among controls over the 6-month period.

### Changes in anthropometry

Mean BMI, WHR and blood pressure scores at 6- ﻿months did not differ from baseline for either group. However, a 0.5 kg reduction in mean weight was evident among the intervention participants from baseline to post-program (*p* = 0.027), whereas no change was recorded in the control group. Table 2 summarises between-group comparisons of all outcomes.

**Table 2:** Comparison of outcomes intervention and controls at baseline and post-program

Outcomes	Intervention group		Control group		
	(*n* = 139)		(*n* = 141)		
	Baseline	Post	Baseline	Post	
Weight: mean (SD) kg	75.57	75.07	76.84	76.86	*p*_2_ = 0.515
	(14.97)	(14.78)	(17.36)	(17.67)	*p*_3_ = 0.359
	*p*_1_ = 0.027		*p*_1_ = 0.924		
Body mass index: mean (SD) kg/m^2^	28.38	28.31	28.62	28.59	*p*_2_ = 0.688
	(4.59)	(4.97)	(5.63)	(5.74)	*p*_3_ = 0.667
	*p*_1_ = 0.660		*p*_1_ = 0.669		
Waist-to-hip ratio: mean (SD)	0.89	0.89	0.89	0.90	*p*_2_ = 0.910
	(0.09)	(0.09)	(0.09)	(0.09)	*p*_3_ = 0.254
	*p*_1_ = 0.727		*p*_1_ = 0.093		
Systolic blood pressure: mean (SD) mmHg	141.01	141.39	141.26	142.42	*p*_2_ = 0.909
	(18.17)	(18.92)	(18.30)	(17.88)	*p*_3_ = 0.640
	*p*_1_ = 0.797		*p*_1_ = 0.434		
Diastolic blood pressure: mean (SD) mmHg	78.40	77.22	78.28	79.23	*p*_2_ = 0.915
	(9.57)	(9.57)	(9.55)	(10.45)	*p*_3_ = 0.096
	*p*_1_ = 0.089		*p*_1_ = 0.199		
Walking time: mean (SD) minutes per week	239.72	231.59	216.01	224.45	*p*_2_ = 0.510
	(324.68)	(260.27)	(275.02)	(270.35)	*p*_3_ = 0.822
	*p*_1_ = 0.769		*p*_1_ = 0.712		
Sitting time: mean (SD) minutes per week	2398.13	2223.38	2426.40	2463.25	*p*_2_ = 0.812
	(905.68)	(1089.16)	(1074.20)	(1103.47)	*p*_3_ = 0.069
	*p*_1_ = 0.064		*p*_1_ = 0.697		
Moderate activity: mean (SD) minutes per week	139.79	219.53	129.18	137.83	*p*_2_ = 0.683
	(165.88)	(280.90)	(257.86)	(174.48)	*p*_3_ = 0.004
	*p*_1_ = 0.003		*p*_1_ = 0.692		
Vigorous activity: ^a^ n (%)	23	27	17	25	*p*_2_ = 0.283
	(16.5%)	(19.4%)	(12.1%)	(17.7%)	*p*_3_ = 0.716
	*p*_1_ = 0.532		*p*_1_ = 0.181		
Strength exercise: ^a^n (%)	33	67	39	42	*p*_2_ = 0.453
	(23.7%)	(48.2%)	(27.7%)	(29.8%)	*p*_3_ = 0.002
	*p*_1_ < 0.001		*p*_1_ = 0.693		
Fruit intake: ^b^n (%)	96	104	92	84	*p*_2_ = 0.497
	(69.1%)	(74.8%)	(65.2%)	(59.6%)	*p*_3_ = 0.007
	*p*_1_ = 0.286		*p*_1_ = 0.325		
Vegetable intake: ^b^n (%)	126	127	123	118	*p*_2_ = 0.363
	(90.6%)	(91.4%)	(87.2%)	(83.7%)	*p*_3_ = 0.052
	*p*_1_ = 0.834		*p*_1_ = 0.398		
Fibre intake score: mean (SD)	3.51	3.60	3.48	3.50	*p*_2_ = 0.715
	(0.58)	(0.55)	(0.63)	(0.56)	*p*_3_ = 0.138
	*p*_1_ = 0.006		*p*_1_ = 0.616		
Fat intake score: mean (SD)	3.50	3.59	3.58	3.63	*p*_2_ = 0.162
	(0.51)	(0.52)	(0.47)	(0.44)	*p*_3_ = 0.527
	*p*_1_ < 0.001		*p*_1_ = 0.092		
Fat avoidance score: mean (SD)	3.65	3.70	3.51	3.62	*p*_2_ = 0.195
	(0.85)	(0.88)	(0.94)	(0.91)	*p*_3_ = 0.444
	*p*_1_ = 0.290		*p*_1_ = 0.049		

*SD* standard deviation

^a^participation of at least 10 min

^b^consumption of at least two servings on 3 to 7 days per week

*p*_1_: *p* value for baseline versus post

*p*_2_: *p* value for baseline intervention versus baseline control

*p*_3_: *p* value for post intervention versus post control

### Regression analysis

Table 3 presents results from mixed regression analyses which confirmed a marginal improvement in weight (*p* = 0.047) and a significant increase in engagement in strength exercise (*p* < 0.001) across the intervention relative to controls. There was also an increase in time spent on moderate-intensity physical activity for both groups at post-test (*p* = 0.011), yet the intervention group committed significantly more minutes per week than controls (*p* = 0.008). The likelihood of frequent fruit intake significantly increased in the intervention group at post-test (*p* = 0.012) relative to controls, whereas no change was apparent in vegetable or fibre intake, fat intake and fat avoidance scores for both groups (*p* > 0.10).

**Table 3:** Mixed regression analysis of outcomes before and after intervention (*n* = 280)

Outcomes	Group^a^ (intervention versus control)			Time^a^ (post versus baseline)			Group × time^a^		
	COE	SE	*p*	COE	SE	*p*	COE	SE	*p*
Weight^b^	−1.30	1.61	0.423	0.02	0.20	0.914	−0.54	0.27	0.047
Body mass index^b^	−0.08	0.60	0.893	−0.04	0.08	0.651	−0.03	0.17	0.855
Waist-to-hip ratio^b^	−0.01	0.01	0.468	0.01	0.01	0.143	−0.01	0.01	0.243
Systolic blood pressure^b^	−0.25	2.21	0.910	1.01	1.36	0.457	−0.79	2.41	0.743
Diastolic blood pressure^b^	0.57	1.28	0.658	0.92	0.52	0.077	−2.16	1.01	0.032
Walking time^bc^	0.12	0.14	0.379	−0.02	0.17	0.909	0.23	0.20	0.257
Sitting time^d^	0.02	0.04	0.558	0.02	0.04	0.640	−0.11	0.05	0.027
Moderate activity^d^	0.96	0.36	0.008	0.44	0.17	0.011	0.07	0.21	0.726
Vigorous activity^e^	0.34	0.36	0.347	0.47	0.34	0.170	−0.26	0.40	0.513
Strength exercise^e^	−0.26	0.30	0.387	0.12	0.19	0.515	1.14	0.31	<0.001
Frequent fruit intake^e^	0.24	0.25	0.335	−0.30	0.19	0.118	0.63	0.25	0.012
Frequent vegetable intake^e^	0.42	0.36	0.241	−0.30	0.23	0.190	0.39	0.45	0.378
Fibre intake score^b^	0.05	0.09	0.588	0.02	0.04	0.545	0.07	0.05	0.189
Fat intake score^b^	−0.05	0.05	0.340	0.05	0.03	0.106	0.04	0.04	0.285
Fat avoidance score^b^	0.13	0.12	0.266	0.12	0.05	0.023	−0.06	0.07	0.418

*COE* coefficient, *SE* standard error

^a^adjusted for age (years), height, gender, education level (secondary school or below, trade certificate/diploma, university), relationship status (no partner, with partner), smoking status (never, former/current) and co-morbidity (no, yes)

^b^linear mixed regression model

^c^logarithmic transformed

^d^gamma mixed regression model

^e^logistic mixed regression model

## Discussion

### Recruitment and retention

This study investigated the impact of a semi-tailored physical activity and nutrition intervention on adults aged 60 to 80 years living in retirement villages, an increasingly popular residential choice for the aging population. The program’s retention rate was high (77.7%) and compared well with similar community-based programs [26, 37]. However some community-based programs for older adults have reported attrition rates of 50%, suggesting this population may be more prone to attrition [30]. Aspects of program tailoring, increasing acceptability and access [38] should be considered as loss of participants may introduce bias and reduce the representativeness of findings. It was reassuring that the demographic characteristics of dropouts in this study did not differ from those of program completers.

The initial recruitment of the retirement villages was challenging as only 38 of the 80 village managers contacted agreed to participate. This was despite several personal contacts made by telephone to explain the study purpose, the feasibility of residents participating and their preferred process for contacting residents. Over half (*n* = 42) of the retirement village managers chose not to take part, despite the program being free, managed by trained professionals and readily available to residents. Further investigation into reasons for their rejection is important in order to optimise response rates in future replications of the program.

This research also highlighted the key role retirement village manager’s play as ‘gate keepers’. The support of managers for recruitment information sessions was essential, with the majority of participants (66.4%) being recruited via this approach. Involvement by managers may have led to perceived ownership of the intervention and therefore more promotion and ongoing support during the study period, thereby increasing program adherence and the likelihood of long-term sustainability after external funding ceased. Independent living is considered a core value of retirement villages [16] and our program was developed to support this core value.

### Physical activity

By the end of the 6-month program, the intervention group showed significant improvements in moderate-intensity physical activity, a mean increase of ~80-min per week. This equated to an extra ~11-min per day and exceeding the recommended physical activity guidelines of 150-min per week for health benefits [27]. Consistent with previous research, very few participated in vigorous-intensity activity [26]. The increase in moderate-intensity physical activity compares favourably with similar physical activity programs for older adults [26, 39] and further demonstrates the substantial gains possible via a low-dose semi-tailored intervention. The retirement village setting may offer even greater potential to influence behaviour change through the use of ‘social norms,’ due in part to the close living proximity of residents within these gated communities. For example, if a culture of involvement in regular walking was seen, it might encourage other like-minded residents to participate and became more active. Further research is recommended to better understand how social norms can be utilised to support positive health behaviours in this setting [40].

Participation rate in strength exercise had doubled at post-test in the intervention group, in contrast to a small (2%) increase in the control group. Participation in strength exercises has the potential to provide many benefits to aging adults including a contribution to muscular strength and endurance with likely improvement in mobility and reduction in the risk of falls and related injuries [41]. Strength exercises were carried out with low-cost resistance bands and body weight exercises, acceptable to the target group and relatively safe [26, 42]. The combination of strength exercises and aerobic activity has the potential to maximise positive health effects [43] and should be considered for any community-based program.

### Dietary behaviours

Lack of knowledge about foods and changing dietary needs may influence the types of foods consumed [4, 44, 45]. This intervention aimed to increase knowledge of appropriate food types and meals through the provision of educational resources and motivational interviewing to support behaviour change. It appears to have had a positive impact in certain dietary behaviours, with the intervention group significantly increasing frequency of fruit intake across the program, along with fibre intake and fat avoidance. These changes are moderately encouraging as many older Australians are not aware of their nutritional needs [4]. However, there was no statistically significant change in vegetable intake, which may require further investigation. These participants may already maintain adequate vegetable consumption, which may have reduced the opportunity for dietary gain [24].

### Weight loss

The 0.5 kg mean reduction in body weight of the intervention group was also encouraging and the adoption of a diet high in fruit and vegetables and low in fats is associated with a lower BMI in older adults [7]. Weight reduction in older adults may be unhealthy if skeletal muscle tissue as opposed to adipose tissue is preferentially lost. We are unable to quantify changes in tissue composition however participants in this intervention were involved in a balanced combination of strength exercises, aerobic activity and dietary education. Considering that approximately 60% of the target population are overweight or obese [46], attempts to improve body composition should involve maximising fat loss and preserving lean tissue and thereby supporting functional and independent living.

### Limitations

The present intervention was limited to 6-months in duration due to budget and resource restraints, however it is comparable to other programs for older adults in the literature. This time period may be considered adequate to reflect changes in behaviour [47], taking into account the likelihood of participant attrition over a more extended period, however. Nevertheless, longer duration studies are recommended for future interventions to determine program sustainability and effectiveness. The inherent self-selection bias could not be avoided for our voluntary participants but was controlled partially through the cluster-randomisation process. Another limitation concerned the lack of objective physical activity and dietary assessments, despite self-report data deemed to be appropriate for determining behavioural changes over time [48], this method can result in over and under-reporting, recall bias and participant burden [49].

## Conclusions

Retirement is a period of time that offers important opportunities for individuals to reassess their lifestyle and adopt health-enhancing behaviours such as positive changes to physical activity and diet. The physical activity and nutrition program outlined was successful in improving body weight, engagement in strength exercises, increasing levels of moderate-intensity physical activity and consumption of fruit among retirement village residents. These behaviour changes, if maintained, may contribute to delaying the onset of chronic diseases, support functional mobility and independent living, a core value of retirement villages. Strategies that enhanced such positive behaviour changes included individual knowledge, skill building and access to appropriate services and facilities. This is important information for those involved in the management of retirement villages, a growing segment of the aging residential population. However, more effective engagement of retirement villages and their managers remains a challenge in future research.

## Additional file

Additional file 1: CONSORT 2010 checklist of information to include when reporting a randomised trial. (PDF 136 kb)

## Abbreviations

BMI: Body mass index
ILU: Independent living unit
kg: Kilogram
WHR: Waist hip ratio
