# Erratum to: Effects of a physical activity and nutrition program in retirement villages: a cluster randomised controlled trial

**DOI:** 10.1186/s12966-017-0568-x

**Published:** 2017-09-21

**Authors:** Jonine Jancey, Anne-Marie Holt, Andy Lee, Deborah Kerr, Suzanne Robinson, Li Tang, A. S. Anderson, Andrew P. Hills, Peter Howat

**Affiliations:** 1 Collaboration for Evidence, Research and Impact in Public Health, School of Public Heath, Curtin University, GPO Box U1987, Perth, WA 6845 Australia; 2 School of Public Health, Curtin University, GPO Box U1987, Perth, WA 6845 Australia; 3 Chinese Evidence-based Medicine Center, West China Hospital, Sichuan University, No. 37 Guoxue Alley, Chengdu, Sichuan Province 610041 China; 4 Centre for Public Health Nutrition Research, Division of Cancer Research, Ninewells Medical School, Dundee University, Level 7, Mailbox 7, Dundee, DD1 9SY UK; 5 University of Tasmania, 41 Charles St, Launceston TAS, Launceston, TAS 7250 Australia

## Erratum

Unfortunately, the original version of this article [1] contained an error. The middle initial of “Andrew P. Hills” was omitted. The original article has been corrected.
